# An empirical analysis of electricity use and expenditure in farming households in Poland

**DOI:** 10.1038/s41598-025-22762-0

**Published:** 2025-11-06

**Authors:** Arkadiusz Piwowar

**Affiliations:** Faculty of Economics and Finance, Wroclaw University of Economics and Business, 53-345 Wrocław, Poland

**Keywords:** Farming household, Electricity expenditure, Rural area, Agriculture, Multiple correspondence analysis (MCA), Energy science and technology, Engineering, Environmental sciences, Environmental social sciences

## Abstract

The article presents the results of empirical research into expenditure on electricity and the dependencies of the share of these expenses with regard to the features of farming households in Poland. The source material came from empirical research conducted on a random sample of 480 farming households in Poland (each exceeding 5 ha of UAA), with multiple correspondence analysis (MCA) used in the analyses. Through a combination of survey methods and MCA, this study aims to assess electricity usage in farming households, with particular emphasis on identifying the portion of energy costs directly linked to agricultural operations. Statistical analysis demonstrated the existence of strong dependencies between the share of expenditure on electricity from agricultural production and the economic size of a farm (φ^2^ = 0.2655), the district (φ^2^ = 0.2561), and the agricultural production system (φ^2^ = 0.1070). The research shows that expenditure on energy constitutes a considerable percentage of total expenses on energy in the studied farming households. The research results may become a point of reference for other techniques and tools used in energy measurements at the micro-economic level, including the combining of various approaches and the modifying of techniques and tools developed earlier. The results can also be an important source of information for the economic and institutional sphere, including operators on the electricity market.

## Introduction

Demand for energy is strongly correlated with economic growth and development in all spheres of social and economic life^1,2^. Socio-economic progress is connected to an increased demand for electricity, which results from the use of more advanced technologies in all sectors of the economy, and the changing needs and habits in households. In terms of households, access to stable sources of electricity at an affordable price is a fundamental need. This makes it possible to ensure the necessary living conditions (lighting, heating, cooling), and the possibility for members of the household to develop in all aspects of social development. The lack of such possibilities is described in the subject literature as energy poverty^3–5^. In terms of the economic sphere, physical and economical access to electricity makes it possible to exploit technical advancements (robotization, automation, etc.), which is fundamental to an increase in the efficiency of business activity. Energy poverty is a critical issue addressed by the United Nations’ Sustainable Development Goals (SDGs). Specifically, Goal 7 aims to “ensure access to affordable, reliable, sustainable, and modern energy for all^6,7^. According to the IEA’s most recent study, “World Energy Outlook 2022”, Europe has been engulfed in an energy crisis since the conflict between Russia and Ukraine, and energy costs have increased. In Western economies, including the US, UK, Eurozone, and Japan, the high cost of energy has impacted other sectors of the economy, leading to increased inflation. The research also notes that food insecurity has worsened in many developing nations due to rising energy prices^8^.

Energy is a crucial production resource in agriculture, used to power machinery and devices in the sector^9–11^. The annual energy use in EU open-field agriculture is at least 1431 PJ (3.7% of total EU annual energy consumption)^12^. The subject of interest of this paper is electricity consumption in farming households in Poland. In a broad scope, the role of energy in agriculture is crucial not only from the point of view of determining the size and quality of agricultural production, but also from the point of view of the quality of the natural environment. With regard to the latter, the fundamentals of the process of generating electricity (raw materials) are of principal importance. In terms of the spatial scope of analyses (Poland), this problem has been widely analysed in the subject literature. Here, considerable progress is underlined in terms of the spread and implementation of renewable sources of energy. Amongst others, Poland has huge unexploited potential for the production of agricultural biogas^13,14^. The growth in demand for electricity in rural areas constitutes significant potential for the development not only of dispersed energy production and the use of renewable energy sources in the form of biomass, but also solar energy, small hydroelectric installations and wind turbines^15^. Nevertheless, it is underlined in the international literature that in recent years there have been far-reaching changes in energy use in agriculture and rural areas^16^.

The issues addressed in this paper relate to estimating electricity use and expenditure on electricity for agricultural purposes in farming households. It should be underlined that agricultural activity is highly sensitive to the aspect of costs related to production factors, including electricity^17–19^. Despite significant progress in energy efficiency in Polish agriculture since the country’s accession to the EU, there is still a need for continued efforts to support the sector’s transition towards more sustainable and energy-efficient practices^20^. Due to the fact that in Poland almost all farms belong to the category of individual farms, the basic group as well as the basic subjective scope are farming households. According to the definition, these are farms where the exclusive or principal (prevailing) source of income is revenue from the use of individual farms for agricultural purposes. According to the results of the General Agricultural Census (PSR 2020), the number of farms in Poland in June 2020 was 1,317,400. Of these, 99.4% (1,309,900) were individual farms owning 91.3% of all agricultural land and 90.8% of all large livestock held on farms. In the year of the study, 96.8% of all full-time employees (counted in AWU^1^) employed in agriculture worked on individual farms.

**Fig. 1:**
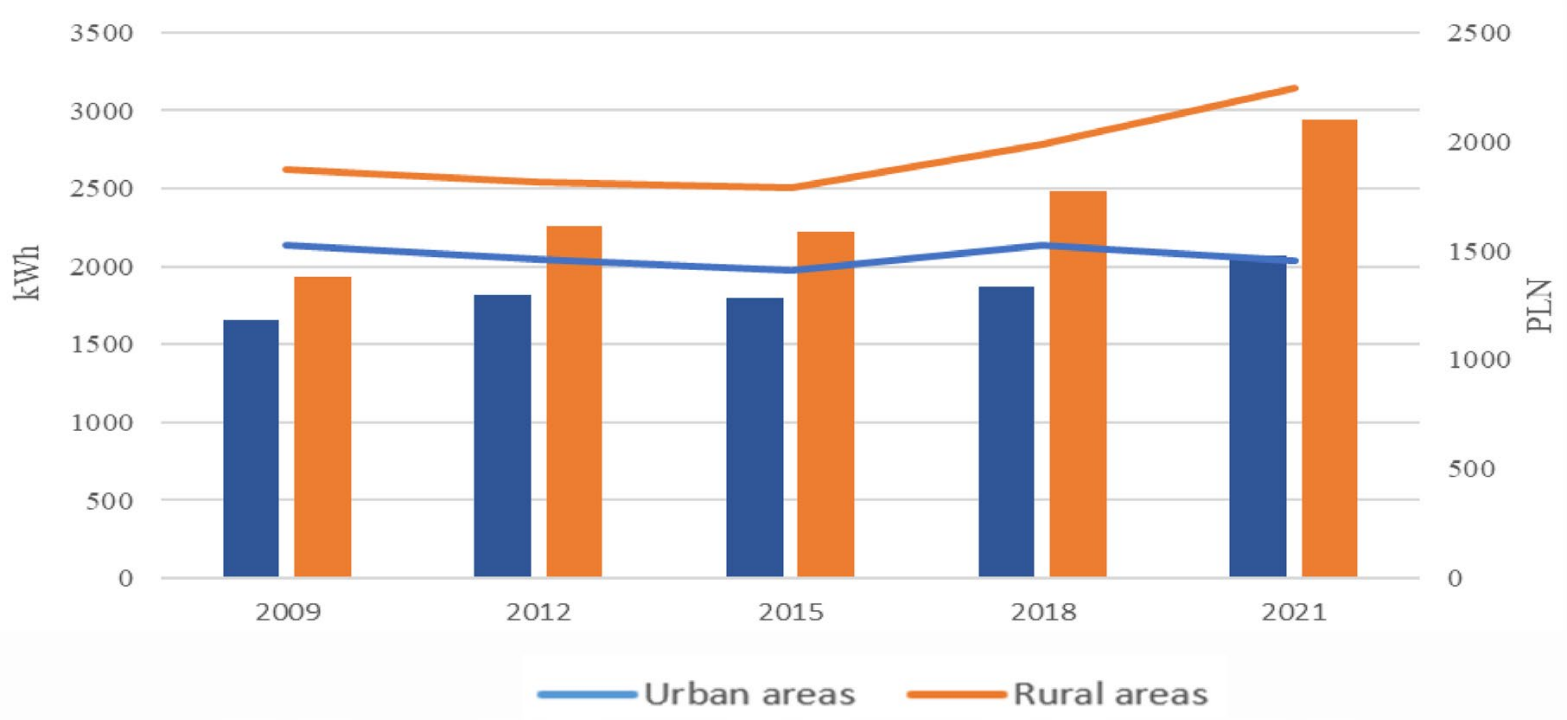
Average yearly electricity usage (kWh, line graph) and expenditure (PLN, bar graph) in urban and rural households in Poland in 2009–2021^22^.

Interest in the issue of expenditure on energy is important both from a theoretical and a practical point of view. Energy takes up a considerable share of production costs in agriculture, and expenditure on energy is the basic cost of agricultural activity. In every instance, the use of technical devices in agriculture can be considered from the point of view of the energy required to carry out the work, as well as its purposefulness and efficiency. It is worth underlining that new economic systems, as well as new organizational systems implemented in agriculture (incl. with regards to precise agriculture), are based on the use of electrical energy. However, in order to determine the efficiency of energy use, it is necessary to monitor and measure such energy use, which in the case of individual farms in Poland is not an easy task. These difficulties are due to the fact that the decided majority of farms do not have a separate meter indicating energy use for agricultural purposes. Additionally, the lack of possibility to obtain data in the desired format from operators on the fuel and energy market, or from other entities, gives rise to the need to address energy consumers directly (e.g. through study questionnaires). As underlined by Kowalczyk-Juśko and Kościk^21^, it is recommended in this type of research to verify the results obtained based on survey questionnaires conducted in one specific territorial unit. The authors emphasize that estimates to date may have a large degree of error, as agriculture in individual voivodeships varies significantly, and such variations maybe even greater at the district level. Thus, the current approach does not meet expectations with regards to analysis of farms and agricultural systems in the sphere of electricity usage. These problems can at least partially be attributed to misunderstandings as to the role of energy in agricultural systems, and the appropriate assigning of expenditure to the cost of living and production purposes. This is all occurring in a situation in which a clear increase in energy use is noted in rural areas in Poland (Fig. 1).

The data presented in Fig. 1 is public statistical data published every 3 years in Poland (the paper presents the latest data made available in May 2023). Nevertheless, the period of time covered by this data corresponds to the time frame of the empirical research presented further in this paper. The data shows that the increase in electricity usage in rural areas was considerably higher than the increase in usage overall, and was also higher than the change in usage in cities.

The topic of this research was chosen due to the lack in publicly available sources of data to figures on the amount of electricity usage in farms in Poland, and also due to the dynamic increase in this respect in rural areas. The principal aim of the article is to determine the level and proportion of farming household electricity expenditure used in family households and agricultural production in relation to farm characteristics (production system, size, and location). Investigating electricity usage in Polish farms is relevant and timely, especially amidst dynamic increases in rural areas. It is worth noting that Poland consistently ranks among the countries with the highest electricity prices in Europe. More research is needed on how to best assist these households with their energy burdens and to provide them with the information and motivation to help improve their situation.

To summarize, the main contributions of this study include: (1) investigating the proportion of electricity expenditure allocated to household use versus agricultural production in Polish farming households, offering insights into energy distribution across domestic and agricultural activities, (2) demonstrating the analytical value of multiple correspondence analysis (MCA) in uncovering complex interdependencies between energy costs and farm characteristics such as size, location, and production system, (3) proposing future research directions, including the integration of seasonal energy usage patterns and the incorporation of socio-economic variables to deepen understanding of household-level energy decisions.

This paper is structured as follows. The introduction is followed by the presentation of an outline of prior findings on the research topic on the basis of published scientific papers. This is followed by a presentation of the research methodology, a chapter with the research results and a discussion. The last part formulates the most important conclusions of the study, as well as proposals for further research directions on the topic.

## Literature review

There are significant issues with measuring energy use related to agricultural infrastructure accurately. These issues often stem from the complexity of energy flows within farms, the lack of separate metering systems for agricultural-specific usage, and the variability of farm types and practices. There is lack of systematic research on electricity use in farm households. This gap in research can make it challenging to develop effective policies and strategies for improving energy efficiency and sustainability in the agricultural sector. In many cases, farming households’ energy systems are used for domestic and agricultural purposes. Inefficient energy use at the household level can impact overall farm efficiency and environmental sustainability. Developing energy policies that simultaneously address household and agrarian needs can create a more unified approach to sustainability, reducing overall environmental impacts and promoting rural development. Agriculture requires energy in various forms for different purposes, making accurate measurement complex^23^.

In light of growing energy expenses and worries about greenhouse gas emissions, on-farm energy use is becoming more and more significant. Energy inputs are essential resources in agricultural production. While they are not expenses in themselves, the associated costs of acquiring and utilizing these inputs significantly impact farmers’ budgets. The lack of reliable, country-specific data often forces researchers to use values from other countries, which may not accurately reflect local circumstances^24^. In the agricultural sector, energy efficiency takes on additional significance due to the often-challenging balance between revenues and costs associated with farming activities^25^. Addressing these challenges requires a concerted effort to improve data collection methods, invest in research, and develop tailored solutions that consider local conditions and needs. The solutions to address challenges or improve energy efficiency often need to be tailored to local needs because of the diverse agricultural and socio-economic contexts^26^.

The issue of energy usage in agriculture and the related costs is an important topic of research papers in the field of agricultural economics. These issues have been considered on many levels, both micro- and macro-economic. From a micro-economic perspective, analyses are conducted into the size and amount of electricity usage, the effect of the variability in the price of electricity on the productivity and efficiency at the farm level, etc. Many research papers address optimizing energy-saving processes in agricultural production, which is key to improving productivity and limiting the negative impact on the natural environment. Analyses in this regard have been conducted for both specific crops^27–29^, as well as for farms situated in specific locations (district, voivodeship, region, etc.)^30^. Meanwhile, macro-economic analyses relate to the entire agricultural sector. Assessment is made of the energy efficiency of the agricultural sector, as well as individual branches of the agro-industrial sector. Analyses in this regard are generally conducted on the basis of secondary data gathered by administrative bodies. One example are analyses based on data available in the Farm Accountancy Data Network (FADN). Such research is conducted in the system of agricultural types, according to physical area, economic size, etc.^31,32^. Analyses are conducted in such a way with regards to the energy consumption structure in agriculture in total, and the impact of energy usage on the overall economic results of farms and various groups of farms, usually using econometric techniques^25^.

Electricity represents a vital component of the total energy used in the agricultural sector. It is often transformed into various forms to power essential processes, such as irrigation systems, crop drying, greenhouse heating, and the operation of machinery and equipment. Its role within the broader context of total energy highlights the importance of analyzing energy usage patterns to ensure efficiency and sustainability in agricultural operations. It is worth maintaining a broader perspective of overall energy use in agriculture. In the case of analyses for Poland, the increase in energy costs are emphasized in relation to both general farming costs as well as indirect usage^33^. Agriculture uses energy both directly in the form of electricity and fuel in crop production, animal products production and transportation of farm productions, and indirectly through use of energy-intensive inputs, most notably fertilizers, seeds and pesticides^34^. For example, the manufacturing of nitrogen fertilizers requires significant amounts of natural gas. Compared to the EU, Polish agriculture uses relatively large amounts of mineral fertilizers, especially nitrogen fertilizers^35^. The amount of energy costs and their share in total costs are strongly dependent on the type of farming. Golasa^36^ showed that in horticultural farms, energy costs are of the greatest importance, constituting as much as 33% of all incurred costs. In the remaining types of individual farms, energy costs constitute from 7.24% (permanent crops) to 15.75% (field crops). Wysokiński et al.^37^ highlighted the unique energy needs of fruit farming in Poland. The interdependency between the value of agricultural production and energy costs has also been determined^38^. This research was conducted on the basis of FADN, while the principal problem in using this data being due to the fact that it only applies to commercial farms^21^. Additionally, the most represented group of farms in the FADN population are small farms (8000 ≤ EUR ˂ 25,000), nearly 80% of the total represented farms. On the other end of the scale are large farms, categorized by standard production exceeding EUR 50,000. Within the FADN population, these farms represent merely 2.4% of all entities included^20^. There are also research results available in the literature referring to evaluation of the energy consumption of Polish agriculture compared to other sectors of the economy on the basis of secondary data from the central statistical office (GUS) and from Eurostat^39^.

Considering the dominating agricultural model in Poland, including the very large number of farms (around 1.3 million), to a large degree of a small area and which do not have a separate electricity meter, this data may not be sufficient for illustrating the actual situation. Not only is FADN data insufficient in this area, but in the scientific literature it is often underlined that the representativeness of the current FADN sample may be inadequate^40^. The FADN database allows in-depth farm activity information to be grasped, but variables (including those referring to energy consumption) are presented on an aggregated level^37^. Taking into account the varying energy usage patterns, which differ not only in individual countries but also due to climatic conditions, the structure of production and energy resources, including within the country^41^, it is necessary to undertake research efforts that aim to determine the level of usage and the factors determining the differentiation in such usage. Additionally, in the area of research, as underlined by Kowalczyk-Juśko and Kościk^21^, the lack of a distinction between the energy used for the two above-mentioned separate purposes creates a problem with estimating the actual energy consumption by farm in a given district. This is also confirmed in the findings of other scientific studies^42,43^.

This paper proposes a different approach to the issue of energy usage at the micro-economic level, and on the basis of study questionnaires (the methodology is described later in the paper), and taking into account the dispersed nature of farms and the varying physical and economic features of farms, estimations are made as to the level of usage and the co-occurrence of categories of variables. Due to the considerable diversity of energy consumers, a reliable assessment of usage required the collection of a large amount of empirical data and the use of multiple correspondence analysis (MCA). This is described in more detail in the methodology section.

## Methodology and data sources

The paper uses both secondary and primary sources of information. The secondary sources include the subject literature and statistical data (GUS) relating to energy usage and expenditure on energy in households in Poland. The principal source of statistical data was a report published by the Central Statistical Office (GUS) under the title “Energy usage in households in 2021” (issued every 3 years).

Meanwhile, the primary sources comprised survey questionnaires conducted in 2019 and 2020 among 480 farming households in Poland. The sampling frame contained macro-regions, and at the first stage of selection, one voivodeship was selected from each macro-region in Poland. The general characteristics of the selected farms is presented in Table 1.

**Table 1:** General characteristics of the test sample.

Poland’s macro-region	Voivodeship	District	Number of farms	Agricultural land area [ha]	Agricultural land area per farm [ha]
Południowo-zachodni	Dolnośląskie	Głogowski	25	911.21	36.45
		Górowski	30	828.51	27.62
		Legnicki	25	586.05	23.44
Północno-zachodni	Zachodnio-pomorskie	Białogardzki	26	2490.30	95.78
		Gryfiński	26	2687.83	103.38
		Koszaliński	28	1131.94	40.43
Wschodni	Lubelskie	Lubartowski	25	461.71	18.47
		Zamojski	28	492.96	17.61
		Bialski	27	730.55	27.06
Północny	Warmińsko-Mazurskie	Giżycki	20	544.65	27.23
		Ełcki	30	1198.84	39.96
		Olecki	30	1285.16	42.84
Południowy	Małopolskie	Nowosądecki	27	219.30	8.12
		Tarnowski	27	420.75	15.58
		Krakowski	26	239.10	9.20
Centralny	Łódzkie	Opoczyński	25	758.20	30.33
		Łęczycki	31	681.96	22.00
		Kutnowski	24	1238.93	51.62
Total			480	16,907.95	35.22

Source: own study based on questionnaire surveys (N = 480).

The combined size of the studied farms amounted to 16,907.95 hectares, of which the largest area was for farms located in the Zachodnio-pomorskie voivodeship. It is worth noting that the Zachodnio-pomorskie voivodeship also stands out against Poland overall due to it having the highest average area per farm (in 2020, it was 31.75 hectares per farm). For the needs of CA (including for legibility in the graphical results elements) the data from districts was merged up to the level of voivodeships.

In the voivodeship system, the largest total area of ​​the surveyed farms was in the Zachodnio-pomorskie and Warmińsko-Mazurskie voivodeships (6310.07 hectares and 3028.65 hectares), the smallest in the Małopolskie voivodeship (879.16 hectares). This is consistent with the total area distribution in Poland. The Utilized Agricultural Area (UAA) varies significantly across Poland’s voivodeships, reflecting regional agricultural practices and land use differences. Zachodnio-pomorskie and Warmińsko-Mazurskie voivodeships typically have larger UAA per farm due to their focus on large-scale farming. Smaller UAA per farm characterizes Małopolskie voivodeship, as these regions have a higher prevalence of small, family-run farms.

Among the studied farms, a slight majority (51.9%) were conducting only crop production. This proportion is similar to the parameters occurring in the general population. Data from the general agricultural census shows that in 2020 in Poland almost 56% of farms conducted exclusively crop production, while 44% conducted both crop and animal production (mixed production), and 0.6% of farms conducted exclusively animal production. Table 2 presents the characteristics of the studied farms in terms of agricultural production system (crop, animal, mixed) as well as the physical size (in hectares) and economic size (in thousands of euros SO). The Standard Output (SO) is a measure used in agriculture to determine the average monetary value of agricultural output at farm-gate prices.

**Table 2:** Production system and physical and economic size of studied Polish farms in 2019. Source: own study based on questionnaire surveys (N = 480).

Specification	Number of responses	Percentage in the research sample [%]	Structure of the general population [%]
Agricultural production system			
Plant production	249	51.88	55.80
Animal production	17	3.54	0.60
Mixed production	214	44.58	43.60
*Total*	*480*	*100*	*100*
Area of agricultural land [ha]			
5–19.99	227	47.29	77.09*
20–49.99	153	31.88	16.88*
50–99.99	68	14.17	6.02*
100 and more	32	6.67	
*Total*	*480*	*100*	100*
Economic size of a farm [SO]			
< 8 thous. euro	91	18.96	64.6
8–25 thous. euro	178	37.08	19.8
25–50 thous. euro	104	21.67	7.8
50–100 thous. euro	71	14.79	4.7
> 100 thous. euro	36	7.50	3.0
*Total*	*480*	*100*	*100*

* without farms less than 5 hectares UAA.

The majority of the farms studied did not exceed 50 hectares of arable land (almost 80% of the research sample). It should be underlined that the agrarian structure in Poland is also dominated by relatively small farms. In Poland overall, farms above 50 hectares constitute only around 3% of the total number of farms. This is a specific feature of Polish agriculture compared to agriculture in the EU-27. In the EU-27, approximately 7.5% of farms are 50 hectares or more in size.

The surveyed sample (n = 480) almost identically reflects the structure of the general population in terms of the agricultural production system. Additionally, the sample featured economically stronger farms, with over 20% of the surveyed farms having an economic size (SO) greater than 50,000 euros. The surveyed sample included more farms larger in area (farms over 20 ha dominated).

**Fig. 2:**
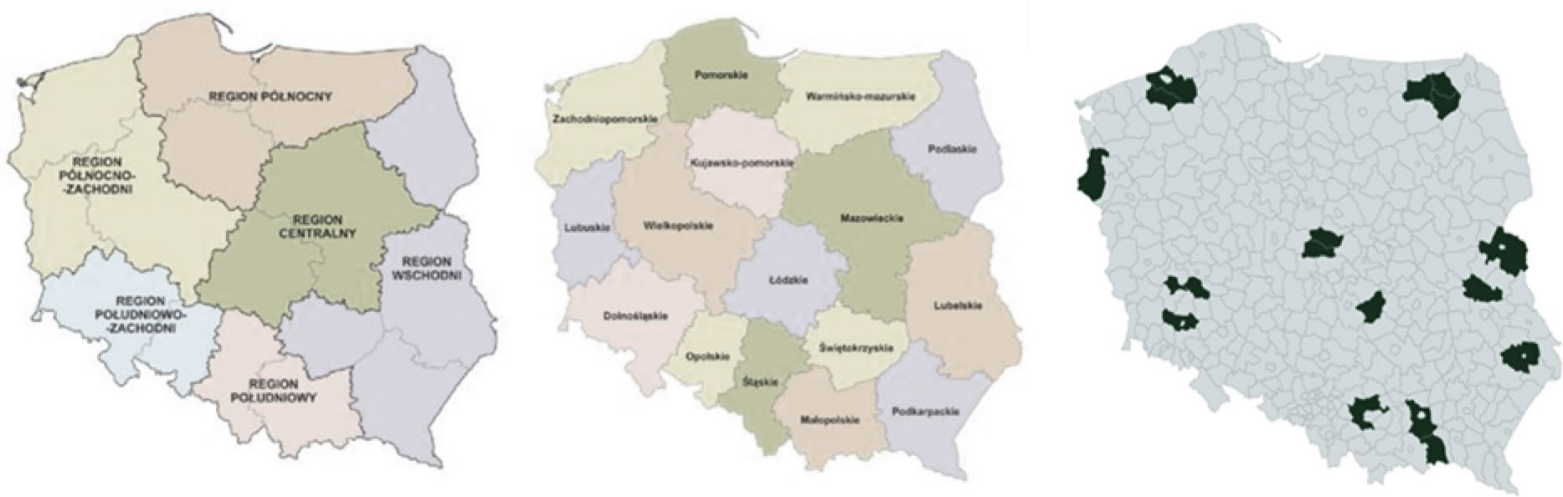
NUTS 1 Macro-Regions (as of December 31, 2017) (**a**), NUTS 2 Voivodeships (**b**), and field study areas in Poland (**c**).

The research was conducted in six randomly selected voivodeships in Poland, and in each voivodeship three districts were selected at random. The research was conducted by employees of agricultural advisory centres, who were employed in teams of advisors in district offices located in the previously selected areas. This was preceded by the concluding of the appropriate agreements for conducting the research process, and the obtaining of permission from the Dean’s Committee for scientific research ethics in the home university. The adopted research assumptions and procedure, i.e. random selection and the conducting of detailed research by agricultural advisors, enabled the collection of high-quality research material. Figure 2 presents the spatial scope of the research with regard to districts in Poland.

The realization of the above research was entrusted to the Agricultural Advisory Centres in the selected voivodeships in Poland, that is:

- The Dolnośląski Agricultural Advisory Centre based in Wrocław,
- The Lubelski Agricultural Advisory Centre based in Końskowola,
- The Łódzki Agricultural Advisory Centre based in Bratoszewice,
- The Małopolski Agricultural Advisory Centre based in Karniowice,
- The Warmińsko-Mazurski Agricultural Advisory Centre based in Olsztyn,
- The Zachodniopomorski Agricultural Advisory Centre based in Barzkowice.

The survey questionnaire, as the principal tool used in this paper, consisted of an extensive set of questions grouped in thematic blocks. The subject of considerations in this article is only the section of the questionnaire relating to expenditure on energy. The remaining sections of the questionnaire were used as the basis for other scientific publications. In the part of the questionnaire entitled ‘Purchase and use of energy and the related expenses’, respondents answered the questions in Table 3 below.

**Table 3:** Survey questions relating to expenditure on energy.

No	Questions	Answers
1	How much of your monthly income do you allocate for electricity bills (please give the total bills for energy—household + farm)?	☐ up to 200 PLN ☐ 200–400 PLN ☐ 401–600 PLN ☐ 601–800 PLN ☐ 801–1000 PLN ☐ above 1000 PLN
2	Please estimate what percentage of the total energy use is for the needs of the farm	☐ up to 20% ☐ 20–40% ☐ 41–60% ☐ 61–80% ☐ above 80%
3	Is there a separate electricity meter for farming activity?	☐ yes ☐ no (please go to question 6)
4	What are the average monthly bills for electricity for the needs of farming activity?	☐ up to 200 PLN ☐ 200–400 PLN ☐ 401–600 PLN ☐ 601–800 PLN ☐ 801–1000 PLN ☐ above 1000 PLN
5	Please give the three most important benefits of having a separate electrical installation and a separate electricity meter for farming activity (select from 1—most important, 2—very important, 3—important)	☐ lower electricity tariffs ☐ the possibility to precisely estimate the costs of farming activity ☐ the possibility to deduct VAT for electricity usage within the scope of farming activity ☐ other (please describe)
6	What are the reasons for not using a separate installation and separate electricity meter for farming activity (multiple choice question)	☐ technical difficulties ☐ I didn’t know about such a possibility ☐ I am not interested due to the high level of fixed fees (for the meter) in relation to the electricity usage for farming purposes ☐ I am not interested due to the unfavourable tariffs for energy usage for farming purposes ☐ I will shortly be shutting down my farm ☐ other (please describe)

The substantive questions were analysed alongside metrics questions relating to the farm: production system, physical and economic size of the farm and its location (district, voivodeship). The principal method of analysis was multiple correspondence analysis (MCA), which enabled the identification and exploration of the links between the study variables. Correspondence analysis (including the multidimensional analysis used in this paper) and its geometric interpretation originated in the 1960s in France and is associated with the French “data analysis’ school”^44–46^. MCA is a analysis method that allows for examining the relationship between two or more qualitative variables in a categorical data set. The foundations of correspondence analysis rests with Pearson’s chi-squared statistic^47^. The correspondence method is widely used for examining data as part of the questionnaire technique, including in social research in the field of agriculture. Correspondence analysis is widely used for analysing response variables (e.g., species variables) and explanatory variables (e.g. environmental variables)^48–50^. It helps to identify patterns and relations between variables and to visualize them in a one- two- or three-dimensional space. In this paper, MCA was used to analyse individual answers to questions relating to the share of energy usage for the needs of farming activity. The basis was to understand the structure of the multidimensional data set, which was the principal aim of this part of the research. Correspondence analysis enabled the assessment of relations occurring between categories of variables measured on non-metric scales, while the φ^2^ mean square multidimensionality index was used to determine the strength of dependencies on the basis of χ^2^ statistic values. These analyses were conducted using Statistica software version 13.3.

## Research results and discussion

### Issues related to measuring energy usage in farming households and tariffs available to farms

**Fig. 3:**
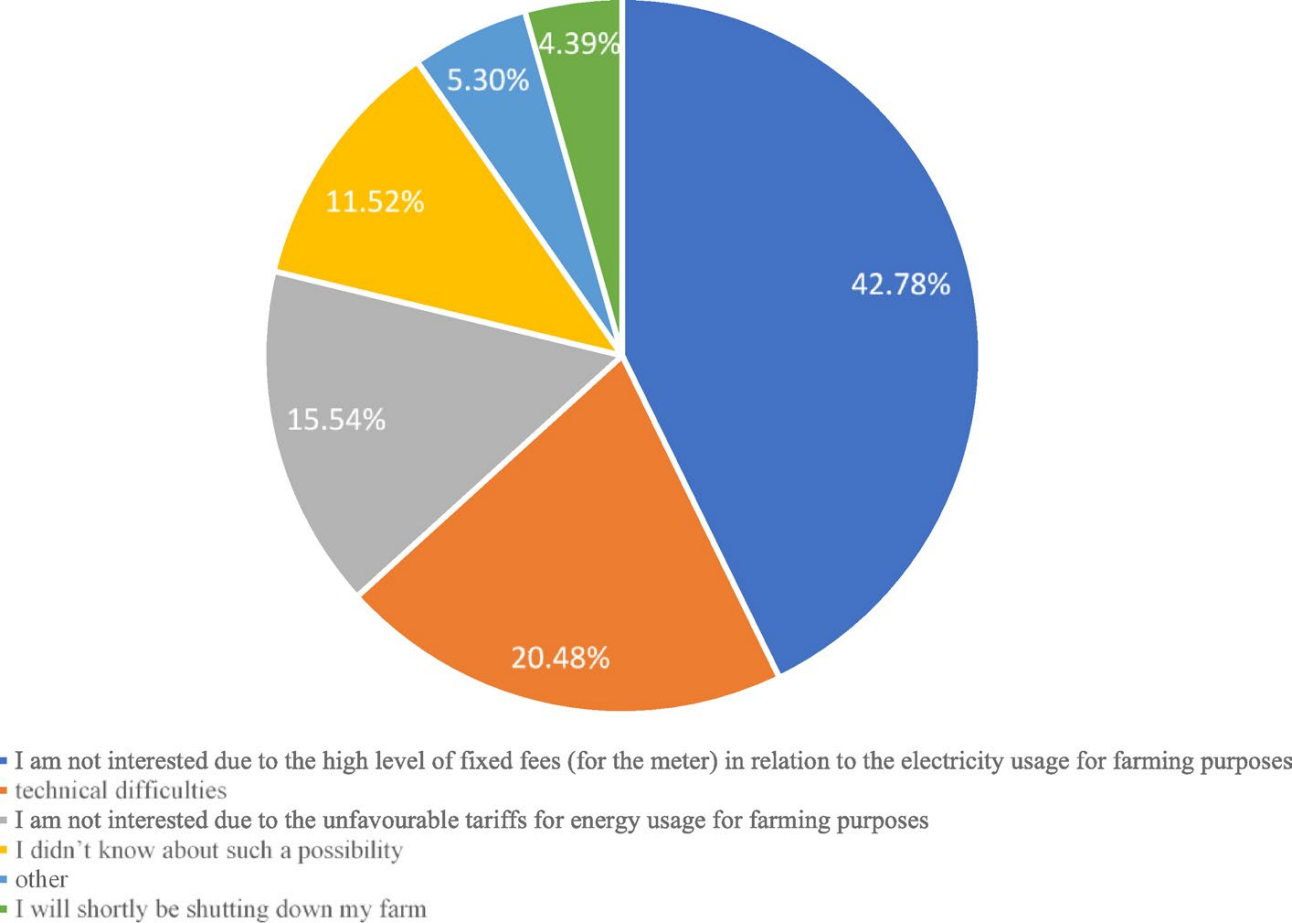
Reasons given by respondents for the lack of interest in installing a separate electricity meter for the needs of farming activity in 2019. *Source*: own study based on questionnaire surveys (N = 425).

As underlined in the introduction, one methodological problem related to the lack of possibility to precisely determine energy usage for farming purposes. Only 55 of the studied farms answered affirmatively to the question about having a separate electricity meter for farming needs. The remaining respondents (i.e. 88.5% of the research sample) did not have such an electricity meter. The respondents were further asked for the reasons why they did not have a separate electricity meter for this purpose, with the results of this analysis presented in Fig. 3.

The respondents had six options to choose from, of which one option was open-ended (other, please describe). In total 547 answers were provided (to the multiple-choice question), of which the answer chosen decidedly most frequently was ‘I am not interested due to the high level of fixed fees (for the meter) in relation to the electricity usage for farming purposes’.

In addition, respondents often pointed to technical problems or unfavourable in their view billing tariffs. Interestingly, one in ten respondents did not know about such a possibility, and 4.59% of answers indicated that the farm would shortly be shut down. From among the other factors, the respondents mainly indicated the relatively low use of electricity for farming purposes.

**Fig. 4:**
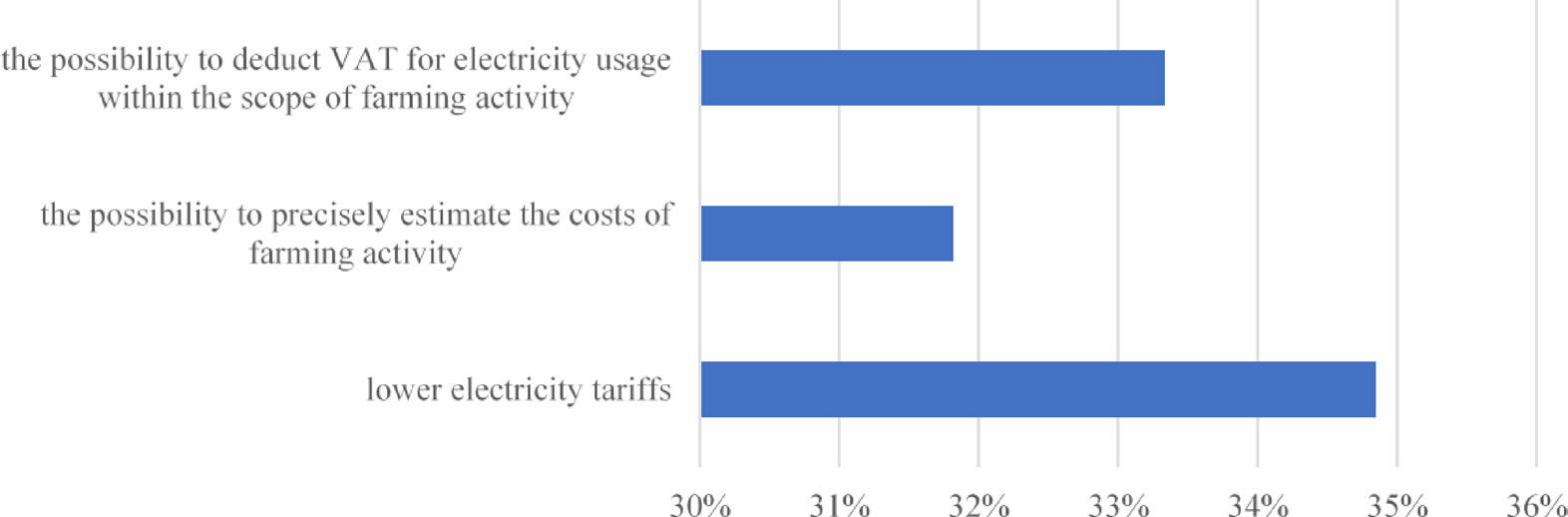
The three most important reasons given by respondents for having a separate electricity meter for farming purposes in 2019. *Source*: own study based on questionnaire surveys (N = 55).

The group of farmers (55 people) who declared that they had a separate electricity meter for farming purposes indicated the most important positive aspects in their view of being in possession of such a solution (Fig. 4).

There is a clear need for an efficient electricity consumption monitoring system that can provide accurate data on farming households energy consumption. The smart meter as a key element for the smart grid is expected to provide economic, social, and environmental benefits for multiple stakeholders^51^. The features of smart meter systems enable farmers to make more informed decisions about energy usage, promoting more efficient and sustainable energy practices. By optimizing energy use, smart meter systems not only reduce operational costs and environmental impact but also contribute to the overall well-being and quality of life for rural communities. As highlighted by recent studies, the integration of smart metering into agricultural settings can foster energy-conscious behaviors, support policy development, and enhance the resilience of future energy systems^52^.

At this point it is worth adding that farmers in Poland have the possibility to be billed separately for electricity usage for agricultural purposes. The distribution of electricity in Poland is carried out by concessioned distribution companies, so-called distribution system operators. These companies are the owners of the distribution networks to which customers, including farms, are connected. There are currently 4 big operators in Poland, i.e. PGE Dystrybucja, Enea Operator, Energa Operator and Tauron Dystrybucja. In addition, there are also 20 or 30 small operators^53^. As underlined in the cited publication, a large proportion of particularly small farms principally conducting crop production are system users in tariff G. However, farmers can choose between tariff G, intended for households and other consumers connected to the low voltage network, and tariff C, which is available for institutional customers, including farms using electricity for production purposes. The selection of appropriate tariff depends on the type and scale of farming activity and on daily energy usage. The offer of sellers is broad and the basic tariffs are:

- with a fixed price for electricity over 24 h;
- with the 24 h divided into several time periods, and the price dependent on the time of day;
- with the 24 h divided into several time periods, and the price dependent on the time of day and day of the week^54^.

### Energy expenditure – size, share, co-occurrence

**Fig. 5:**
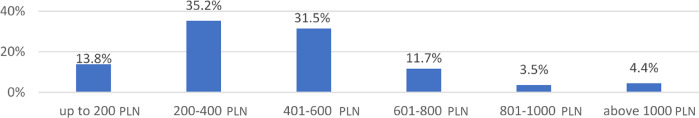
Monthly expenses related to electricity declared by the studied farms (household + farm) in 2019. *Source*: own study based on questionnaire surveys (N = 480).

**Fig. 6:**
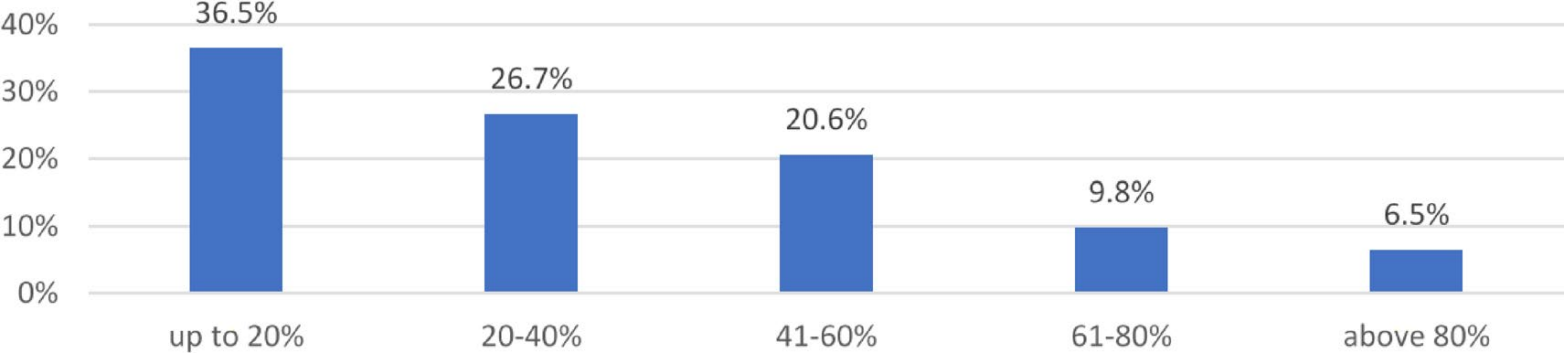
Share of electricity usage declared by respondents for the needs of farming activity in total household usage in 2019. *Source*: own study based on questionnaire surveys (N = 480).

Expenses incurred for purchasing electricity are one of the principal elements in the studied households’ expenditure structure (Figs. 5 and 6). The share of electricity used in the analysis is the farmer’s belief in the share of energy distinction between the farm and household activities.

The research shows that expenditure on electricity varies considerably in the study group. A significant proportion of respondents (35.2% of the research sample) declared monthly expenses of between 200 and 400 PLN (44–88 EUR, 1 PLN is approximately equal to 0.22 EUR). A slightly smaller group (31.5% of the research sample) declared expenses of between 401 and 600 PLN (88.22–132 EUR). This distribution shows a wide range of electricity expenditure shares among the respondents. The differentiation is significant, with a large proportion of households (36.5%) allocating up to 20% of their electricity expenses to agricultural production, while a smaller yet notable proportion (6.5%) allocates more than 80% to agricultural production. These variations can be attributed to several factors, including:

- the economic size of the farms,
- the type of agricultural production system used,
- regional differences in energy costs and availability,
- individual preferences and needs.

The size of electricity bills may also vary depending on the energy supplier. It is worth noting that according to GUS data (on the basis of the E-GD questionnaire), average monthly expenses related to the use of electricity in rural households in Poland in 2021 was 174.90 PLN (estimated on the basis of the number of households using this form of energy i.e. 4,050,066 households). The average yearly household use was 3,147 kWh. However, this data includes all households in rural areas and not only farming households. This information can serve as a useful benchmark for comparing the energy expenses of farming households to the general rural population.

The substantial discrepancy also related to the share of energy usage (and the related expenses) declared by respondents for the needs of agricultural production in total expenditure on electrical energy. The majority of respondents (36.5%) answered that this share was 20%. However, a sizable percentage of replies also showed that costs for farming-related expenses predominated (more than 16% of respondents stated that costs for this purpose exceeded 61% of total expenditures). A more detailed analysis regarding expenditure, taking into account selected variables, is presented in Tables 4 and 5.

**Table 4:** Distribution of the studied Polish farming household according to monthly expenditure on electricity in 2019 (combined household and farm expenditure). *Source*: own study based on questionnaire surveys (N = 480).

Specification	Monthly expenditure on electricity [PLN]					
	Up to 200	200–400	401–600	601–800	801–1000	Above 1000
Area of agricultural land [ha]						
5–19.99	18.5%	40.5%	28.2%	8.8%	1.8%	2.2%
20–49.99	9.8%	32.7%	32.7%	16.3%	5.2%	3.3%
50–99.99	10.3%	27.9%	33.8%	11.8%	5.9%	10.3%
100 and more	6.3%	18.8%	46.9%	12.5%	3.1%	12.5%
Economic size of a farm [SO]						
< 10 thous. euro	21.4%	39.3%	34.5%	2.4%	1.2%	1.2%
10.1–13 thous. euro	23.3%	46.5%	22.1%	4.7%	2.3%	1.2%
13.1 – 20 thous. euro	10.8%	38.6%	34.9%	12.0%	3.6%	0.0%
20.1–50 thous. euro	7.2%	35.1%	31.5%	20.7%	4.5%	0.9%
50.1–100 thous. euro	12.7%	23.9%	31.0%	15.5%	7.0%	9.9%
> 100 thous. euro	2.8%	16.7%	33.3%	13.9%	2.8%	30.6%

**Table 5:** Distribution of the studied Polish farming household according to the share of electricity usage for the needs of studied Polish farming activity in total household usage in 2019. *Source*: own study based on questionnaire surveys (N = 480).

Specification	Up to 20%	20–40%	41–60%	61–80%	Above 80%
Area of agricultural land [ha]					
5–19.99	44.1%	30.4%	17.2%	6.6%	1.8%
20–49.99	30.7%	24.2%	22.9%	12.4%	9.8%
50–99.99	27.9%	25.0%	22.1%	14.7%	10.3%
100 and more	28.1%	15.6%	31.3%	9.4%	15.6%
Economic size of a farm [SO]					
< 10 thous. euro	51.6%	25.8%	19.4%	2.2%	1.1%
10.1–13 thous. euro	47.7%	36.0%	11.6%	3.5%	1.2%
13.1 – 20 thous. euro	34.9%	32.5%	21.7%	9.6%	1.2%
20.1–50 thous. euro	31.5%	27.9%	22.5%	13.5%	4.5%
50.1–100 thous. euro	26.8%	16.9%	29.6%	12.7%	14.1%
> 100 thous. euro	8.3%	8.3%	19.4%	27.8%	36.1%

The analyses show that the relatively high costs and the high share of these costs affected the largest farms in a physical sense (area in hectares) and in an economic sense (economic size of farms). With an increase in the size of farm (especially in the group above 50 hectares), the average expenditure on electricity also grew (over 10% of farms in these groups declared monthly expenditure above 1,000 PLN). High variability was noted in terms of the distribution of the studied farms according to the share of electricity usage for the needs of farming activity in the overall usage in the household, which constituted the basis for conducting more detailed analyses.

Multidimensional correspondence analysis was conducted in order to determine the dependencies and the strength of relations between the studied features. In order to verify whether there are dependencies between the analysed features, a test of independence of nominal features was used based on the χ^2^ statistic. Verification was conducted at a significance level of α = 0.01. On the basis of the χ^2^ statistic values, the strength of the dependencies between the variables was also determined using the mean square multidimensionality index φ^2^ (Table 6).

**Table 6:** Values of the χ^2^ statistic, critical values χ^2^α = 0.01* (in brackets), mean square multidimensionality φ^2^ for features related to the share of expenditure on electricity related to agricultural production, agricultural production system, area of agricultural land, economic size of a farm and location (voivodeship). *Source*: own elaboration.

χ^2^	Agricultural production system c*** = 3	Area of agricultural land c*** = 4	Economic size of a farm c*** = 6	Voivodeship c*** = 6
φ^2^				
Share of expenditure on electricity from agricultural production r** = 5	51.3547 (20.0902)	18.6196 (26.2170)	127.4273 (43.3140)	122.9349 (43.3140)
	0.1070	0.0388	0.2655	0.2561

*Critical values χ^2^α = 0.01 read from the tables for (r − 1) × (c − 1) degrees of freedom.

**Number of rows of the variables analysed.

***Number of columns of the variables analysed.

The critical values read from the chi-square distribution tables, at a significance level of α = 0.01, for three pairs of features are smaller than the calculated χ^2^ statistics. Statistical analysis demonstrated the existence of strong dependencies between the share of expenditure on electricity from agricultural production in the overall expenditure of the studied farms, and:

- the economic size of a farm (φ^2^ = 0.2655),
- the district (φ^2^ = 0.2561),
- the agricultural production system (φ^2^ = 0.1070).

There is a strong dependency between the economic size of a farm and the share of expenditure on electricity from agricultural production. Larger farms are likely to have a higher share of energy costs due to their greater operational needs. Larger farms, due to their extensive operations—such as advanced machinery (e.g. drones used for crop monitoring, pest detection, etc.), irrigation systems, and climate-controlled storage—tend to allocate a higher proportion of their production costs to electrical energy. The district in which a farm is located significantly influences the share of expenditure on electrical energy. This could be due to regional differences in overall cost of electricity beyond the regulated base price, availability of energy sources, and local agricultural practices. While the base price of electricity is regulated, additional costs like transmission and distribution fees may vary regionally. Furthermore, the availability of renewable energy sources, such as solar or wind, can influence energy costs if farms adopt these alternatives. Local agricultural practices, such as greenhouse farming or intensive irrigation, also play a role in shaping electricity usage patterns. The type of agricultural production system (e.g., crop production, livestock farming, mixed farming) also shows a strong dependency on the share of electricity expenditure. Livestock farming, for example, typically involves higher energy use for climate control and automated feeding systems compared to crop production.

**Fig. 7:**
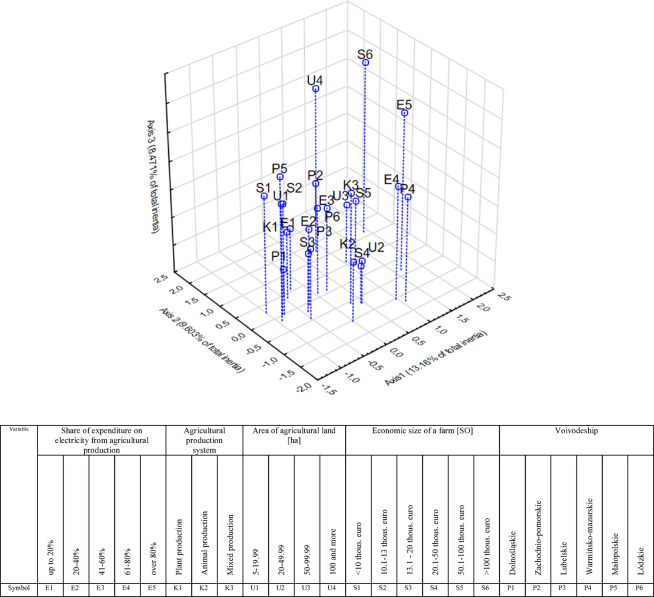
Graphical presentation of correspondence analysis relating to share of expenditure on electricity from agricultural production, and selected production and economic features of the studied Polish farms in 2019. *Source*: own study based on questionnaire surveys (N = 480).

As a result of the correspondence analysis, a geometrical map was generated of this dependency showing the interdependencies between the studied variables (Fig. 7).

The results of the correspondence analysis based on the Burt matrix showed that there is co-occurrence between the studied variables. Respondents who declared a relatively small share of costs for the purchase of electricity for the needs of agriculture (E1, E2) were characterized by a relatively small economic size of farm (S1-S3), a small area of arable land (U1) and the production of crops (K1). On the other hand, there was a characteristic co-occurrence of a high share of expenditure on electricity related to agricultural production in overall expenditure on electricity (E4, E5) with a location in the Warmińsko-mazurskie voivodeship (P4). In this voivodeship, the research sample was dominated by farms with mixed production.

The integration of different types of production increases the complexity and energy requirements of the farm operations^55^. The Warmińsko-Mazurskie Voivodeship is known for its vast agricultural lands and diverse climatic conditions. Warmińsko-Mazurskie Voivodeship is specific in Poland due to its high level of forest cover, numerous lakes, and ecological sites. In the Warmińsko-Mazurskie Voivodeship is a great interest of investors in RE, there is usually a great public support for new energy sources, and the biggest barriers are high investment costs and complicated law in Poland^56^. The region’s climate necessitates the use of more electricity for irrigation, heating, and cooling systems to maintain optimal conditions for crop and livestock production. High energy consumption in dairy production is related to the equipment used and a number of activities: milk cooling system, water heating system, milking machine system, lighting systems, water pump systems and the winter housing facilities^57^.

It is important to exercise caution when interpreting regional differences in energy expenditure. Apparent disparities may not solely reflect the influence of geographical location, but rather stem from structural differences in farm characteristics—particularly physical size and economic scale. For instance, the higher energy expenditures observed in the Warmińsko-Mazurskie Voivodeship may be attributed not to the predominance of mixed production systems alone, but to the fact that farms in this region tend to be larger. Without controlling for these variables, conclusions about regional or production-type effects risk conflating correlation with causation.

Therefore, future analyses should consider comparing farms of similar physical and economic size across regions to isolate the true impact of location and production type. Additionally, the relationship between energy consumption and production type warrants further clarification. Mixed production systems may inherently require more energy due to their operational complexity, but this must be disentangled from the confounding effect of farm scale.

Results obtained in this study are similar to the previous studies. This applies especially to the importance of location features and the related varying environmental factors affecting the amount of energy usage in agriculture^12,58,59^. Agricultural electricity consumption depends on many factors such as irrigation, crop type, season, soil, and land–water level^60^. However, research has underlined the importance of another factor, i.e. the economic size of farms. Due to the varying methods and techniques of agricultural production, the previously accepted measurements taking into account usage per physical hectare should be supplemented with analysis of variations in usage based on economic measurements (e.g. the economic size of farms). Further research directions should make it possible to take into account additional variables characterizing, amongst others, the state of the natural environment (in terms of its impact on electricity usage) and the technical resources at the disposal of farms.

## Conclusions and future directions

Electrical energy usage in farming households is complex due to a variety of interconnected factors. Energy is shared between domestic use and agricultural production, making it challenging to separate and accurately measure these components.

There is no doubt that the basis for precise calculations regarding energy usage in agriculture, as well as energy efficiency and energy consumption, are appropriate measurements and monitoring of electricity consumption. The lack of separate electricity meters makes it necessary to seek research methods that aim to estimate the size and cost of usage. This study investigates the use of questionnaire-based data collection and correspondence analysis to evaluate electricity expenditures in farming households. It focuses on quantifying both the absolute amount and the relative share of electricity costs attributable to agricultural activities within overall household energy expenses. Statistical analysis demonstrated the existence of strong dependencies between the share of expenditure for agricultural purposes in the studied farming households, and the economic size of a farm (φ^2^ = 0.2655), the district (φ^2^ = 0.2561), and the agricultural production system (φ^2^ = 0.1070). Although seldom underlined in the subject literature, of particular importance is the issue of location, including the identification of differences regarding the topic of research in the context of natural conditions and production potential. Furthermore, this research demonstrates that MCA is an important addition to the list of techniques that can be used to identify behaviours related to energy consumption and the estimation of the related costs. Using categorical variables, it is possible through MCA to identify behavioural variability patterns at the individual level within various groups of farms (in terms of area and economic size), as well as between them.

While this study offers valuable insights into household energy expenditure patterns, several limitations should be acknowledged. First, the structural characteristics of the sample, particularly the predominance of farms over 20 hectares, may affect the applicability of the findings to smaller-scale farms. To enhance the analytical depth of future research, it is advisable to normalize energy usage using operational metrics that reflect farm performance and output. Suggested indicators include: electricity expenditure per hectare of UAA; electricity expenditure per PLN of agricultural output; energy consumption per unit of product, such as kWh per kilogram of grain or kWh per litre of milk. These metrics would enable more meaningful cross-farm comparisons, facilitate the identification of inefficiencies and best practices, and support the development of targeted energy policies and support mechanisms tailored to specific farm profiles. It is recommended to expand the set of criteria and alternatives used in the study and to add different social, economic and environmental factors.

This article may be useful for specialists in the field of agriculture, researchers, government officials and all those interested in analyses into electricity consumption in agriculture. The results may be of importance in planning public support systems, especially in the face of sharp price rises on the electricity market. These observations may also constitute useful pointers regarding the issue of investments in renewable sources of energy and the adoption of the role of prosument on the electricity market. In recent years, the role of renewable energy (RE) has gained significant traction in Poland, particularly among family farms and rural households. This shift has been largely driven by financial incentives and national energy policies aimed at decentralizing energy production and promoting sustainability. Among the various RE technologies, photovoltaics (PV) have emerged as the most popular solution, especially in rural areas where energy costs can be a substantial burden on agricultural operations. Additionally, this increases the need to create intelligent measurement and monitoring systems.

Undoubtedly, further more in-depth research is necessary into the daily, seasonal and annual demand for energy at the farm level. This requires the collection of precise data on the type of activity conducted (crops, animal husbandry and breeding). Above all, such research will highlight the problem of the lack of separate data on energy consumption for the needs of agriculture. A considerable number of the respondents did not know about such a possibility, which is an important conclusion for distribution companies with regards to communication. The process of selecting tariffs is complicated due to their complexity, which compounds the need for advice for farmers. Such messages could underline the positive aspects indicated in this research by farmers with separate electricity meters for their farming activity. The research issues can also be applied to a broader context. Progress in this regard, including the development of modern systems for measuring electricity consumption (including intelligent electricity meters in the most important circuits), will make it possible to conduct analysis with regard to possibilities for improvement in energy efficiency, which in turn will enable a reduction in energy requirements in agriculture, lower the costs of farming activity, and reduce the emission of greenhouse gases. Such devices may also be useful for developing an energy consumption profile, useful for optimizing tariffs, taking into account the specifics of the activity of a particular farm.

## Data Availability

The datasets analysed during the current study are available from the corresponding author on reasonable request.

## Abbreviations

AWU: Annual work unit
CA: Correspondence analysis
CEEB: Central register of building emissions
EU: European union
FADN: Farm accountancy data network
GUS: Statistics Poland
IEA: International energy agency
kWh: Kilowatt-hours
MCA: Multiple correspondence analysis
ODR: Agricultural advisory centres
PJ: Petajoule
PSR: Agricultural census
RE: Renewable energy
SO: Standard output
SDGs: Sustainable development goals
UAA: Utilized agricultural area

## Footnotes

1 AWU (Annual Work Unit) is a standardized measure used in agricultural statistics to represent the equivalent of one person working full-time for one year.
